# Movement of Soil-Applied Imidacloprid and Thiamethoxam into Nectar and Pollen of Squash (*Cucurbita pepo)*

**DOI:** 10.1371/journal.pone.0039114

**Published:** 2012-06-27

**Authors:** Kimberly A. Stoner, Brian D. Eitzer

**Affiliations:** 1 Department of Entomology, The Connecticut Agricultural Experiment Station, New Haven, Connecticut, United States of America; 2 Department of Analytical Chemistry, The Connecticut Agricultural Experiment Station, New Haven, Connecticut, United States of America; U. Kentucky, United States of America

## Abstract

There has been recent interest in the threat to bees posed by the use of systemic insecticides. One concern is that systemic insecticides may translocate from the soil into pollen and nectar of plants, where they would be ingested by pollinators. This paper reports on the movement of two such systemic neonicotinoid insecticides, imidacloprid and thiamethoxam, into the pollen and nectar of flowers of squash (*Cucurbita pepo* cultivars “Multipik,” “Sunray” and “Bush Delicata”) when applied to soil by two methods: (1) sprayed into soil before seeding, or (2) applied through drip irrigation in a single treatment after transplant. All insecticide treatments were within labeled rates for these compounds. Pollen and nectar samples were analyzed using a standard extraction method widely used for pesticides (QuEChERS) and liquid chromatography mass spectrometric analysis. The concentrations found in nectar, 10±3 ppb (mean ± s.d) for imidacloprid and 11±6 ppb for thiamethoxam, are higher than concentrations of neonicotinoid insecticides in nectar of canola and sunflower grown from treated seed, and similar to those found in a recent study of neonicotinoids applied to pumpkins at transplant and through drip irrigation. The concentrations in pollen, 14±8 ppb for imidacloprid and 12±9 ppb for thiamethoxam, are higher than those found for seed treatments in most studies, but at the low end of the range found in the pumpkin study. Our concentrations fall into the range being investigated for sublethal effects on honey bees and bumble bees.

## Introduction

The long-term security of insect pollination for food crops is a major concern in the U.S. [1] and around the world [2], [3]. Beekeepers have suffered major losses of honey bee (*Apis mellifera*) colonies annually for the last four years in the U.S. [4], and in parts of Europe [5]. In addition, formerly common species of bumble bees (*Bombus* spp.) have undergone major losses in range in North America [6] and Europe [7]. Many potential factors could be involved in these global declines of managed and wild pollinating insects. For honey bees, losses of managed populations have been attributed to the worldwide movement of parasitic mites, viruses, and the pathogen *Nosema ceranae*; loss of genetic diversity; loss of bee forage; and global trade and economic changes; as well as changes in pesticide use [1], [4], [5]. For bumble bees, losses of species diversity have been attributed to changes in land use with reduced season-long bee forage and nesting habitats, spread of pathogens (*Nosema bombi* and *Crithidia bombi*) from commercial bumble bee colonies to wild populations, and fragmented populations with low genetic diversity, with changes in pesticides use cited as a possible additional factor [1], [6], [7].

Although honey bees are exposed to a wide range of pesticides – including those applied to the hive by beekeepers as well as those in the environment [8] – a class of systemic insecticides known as neonicotinoids has come under particular scrutiny as a result of heavy mortality of honey bee colonies associated with seed treatment of sunflower and corn with imidacloprid in France [9] and seed treatment of corn with clothianidin in Germany [5]. Neonicotinoids include imidacloprid, thiamethoxam, clothianidin, acetamiprid, thiacloprid, nitenpyram, and dinotefuran, and as a group comprise 24% of the global insecticide market [10]. Imidacloprid is the largest selling insecticide in the world, with sales of $1091 million in 2009 and registered for 140 crop uses in over 120 countries [10]. Thiamethoxam is the second largest selling neonicotinoid with sales of $627 million in 2009 and registered for 115 crop uses in at least 65 countries [10]. Neonicotinoids applied to the seed are taken up by the roots and travel through the entire plant to the flowers [10], [11]. Previous field studies measuring the concentration of neonicotinoids in canola, corn or sunflowers, where the seed was treated with the insecticide before sowing, found mean concentrations from 2 to 3.9 ppb in pollen [12]–[14] and from 2.2 ppb to 3.0 ppb in nectar [12], [13]. Two studies using radiolabeled imidacloprid applied to sunflower seed under more controlled conditions found concentrations of 3.9 ppb in pollen and 1.9 ppb in nectar [15] and a concentration of 13±13 ppb (mean ± sd) in pollen [11].

Neonicotinoids are applied to plants in other ways besides direct treatment of the seed [10]. They are applied by foliar spray treatment, by trunk injection in trees, as granules to potting mix or soil, and as liquid sprayed directly to soil or applied through drip irrigation [10], [16]. Little research has been done to quantify the exposure of bees and other pollinators to these pesticides applied in other ways besides seed treatment [16]. Concurrent with our own research, a similar study comparing methods of application of neonicotinoids to pumpkins and measuring concentrations of parent compounds in nectar and pollen was conducted in Maryland [17].

The goal of our project was to quantify movement of two neonicotinoid insecticides into the pollen and nectar of plants when applied directly to the soil, either by direct spray to the soil just before seeding or through drip irrigation. Although we did not quantify bee exposure to these insecticides, knowledge of the neonicotinoid concentrations in the matrices consumed by bees can be compared to those found to have sublethal effects on bees in the scientific literature.

We chose squash (*Cucurbita pepo*) for study because it is routinely treated in the U.S. for control of striped cucumber beetles with systemic insecticides through soil application of neonicotinoids by direct spray to the seed furrow or through irrigation [18]; the flowers are large and both pollen and nectar can be collected in quantities suitable for analysis [19]; and insect pollination is required for fruit set [20], [21]. The major pollinators of squash in the eastern U.S. are squash bees, *Peponapis pruinosa,* a specialist feeding its larvae exclusively on pollen from the genus *Cucurbita* [22], bumble bees (*Bombus impatiens*), and honey bees [20]–[23].

## Materials and Methods

### Planting and Insecticide Application

In 2009, yellow summer squash, *Cucurbita pepo* L. cv. “Multipik,” was grown on black plastic mulch in rows on 1.5 m centers with seed holes spaced at 0.9 m. For the direct-seeded treatments, three seeds were planted per hole. For the transplanted treatments, three seeds per cell were started in the greenhouse before transplanting, and one cell was transplanted per seed hole. Fertilizer (NPK 10-10-10) was applied at a rate of 90 kg/ha of nitrogen, and lime was applied as recommended based on soil tests. The field was laid out in a randomized complete block design with three blocks and five treatments: 1) untreated control; 2) imidacloprid (at 358 g [AI]/ha; Admire Pro®, Bayer Crop Science, Research Triangle Park, NC) applied by surface spray to the soil in the planting hole (11 cm diameter) and immediately incorporated into soil with hand tools, one day before seeding; 3) thiamethoxam (at 140 g [AI]/ha; Platinum®, Syngenta Crop Protection, Greensboro, NC) applied to the soil in the planting hole as above; 4) imidacloprid applied at the same rate per ha as #2 using a Venturi injector through drip irrigation to the entire row five days after transplanting; and 5) Platinum® applied at the same rate per ha as #3 using a Venturi injector through drip irrigation to the entire row five days after transplanting. The chronology of planting, pesticide applications and sampling for 2009 and 2010 are presented in Table S1 of the supplementary material.

In 2010, in a different field where neonicotinoid insecticides had not previously been used, three blocks were planted with yellow summer squash “Sunray F1” and a fourth block was planted with winter squash, *Cucurbita pepo* L. cv. “Bush Delicata.” All five treatments were applied as in 2009, but the rates were different: imidacloprid was applied at 411 g [AI]/ha and thiamethoxam was applied at 143 g[AI]/ha. In both years, the rates of imidacloprid and thiamethoxam applied were within the range of labeled rates (281–420 g[AI]/ha for imidacloprid as Admire Pro® and 89–193 g[AI]/ha for thiamethoxam as Platinum®).

Rainfall was very different during the two growing seasons of the study. In 2009, there were 19.6 cm of rain in June and 16.6 cm in July. In 2010, there were 9.1 cm of rain in June and 9.5 cm. in July. In 2009, no irrigation was used other than the irrigation to apply the insecticides through the drip lines. In 2010, one additional irrigation of the entire field was applied through the drip lines on 8 July.

### Sample Collection

Plant samples were collected over a longer period in 2010 than in 2009 (Table S1) because there was a greater spread among flowering times of the different types of squash (summer and winter), treatments, and even among blocks within a treatment. As female flowers appeared in each plot, they were collected with a clean razor blade, the petals and stigmata were removed, and the remaining bases of the flowers, where the nectaries are located, were saved for chemical analysis. Collection continued in each plot until a 50-ml centrifuge tube was packed full or until all available female flowers from the center row of the plot were collected. Similarly, as male flower buds appeared, the fully developed flower buds were opened before anthesis and the synandria (cone-like male flower structures made of fused anthers) were collected for later chemical analysis.

In 2009, whole-plant samples were taken by randomly selecting a single seed hole from the center row of each plot and collecting all squash plants growing from that hole (generally three plants, but some seed holes had only one or two plants, if not all seed germinated). The total weight of all plant material from that seed hole was recorded.

Nectar was collected with an Eppendorf pipette from female flowers that had been enclosed the previous afternoon in a pollinating bag (Lawson #217, Lawson Pollinating Bags, Northfield, IL). Nectar collection continued as long as female flowers were available in order to get as much nectar as possible for analysis. The nectar from all three blocks was pooled in 2009, and nectar from the three blocks of summer squash was pooled in 2010 in order to have enough nectar for reliable chemical analysis. Nectar from the winter squash in 2010 was collected later and analyzed separately.

Pollen was collected by hand-collecting open male flowers that had been enclosed the previous afternoon in a pollinating bag (as above). Flowers were collected from 6 until 10 am into a large plastic bag, which was then taken back to the laboratory where pollen was scraped by hand, using a thin plastic sheet, from the synandrium of each flower. The plastic bags of flowers were stored for up to one week at 4 C. After pollen was collected, it was stored at –18 C until analysis. In 2009, the pollen was pooled across all three blocks in order to have enough for analysis, but a second sample was taken a week later, also pooled across blocks. In 2010, we collected enough pollen to analyze the blocks separately.

Each plot consisted of 3 rows, and all samples were taken from the center row in order to avoid edge effects. All plant material collected was kept in a cooler on an ice pack during the day of collection and then stored at −18 C until analysis, except for the male flowers for pollen analysis, which were handled as described above.

### Chemical Analysis

**Table 1:** Neonicotinoid insecticide residues observed in 2009 in various tissues of summer squash after application either to the seed hole just before planting (Soil) or to the transplanted plant through drip irrigation (Drip).

	Imidacloprid (ppb ± SD)		Thiamethoxam (ppb ± SD)	
Tissue	Soil	Drip	Soil	Drip
Whole Plant	47±37	218±52	154±44	362±22
Female Flower Bases	10±5	31±17	10±2	22±5
Synandria	15±5	46±4	19±6	31±4

#### Extraction

All samples were extracted using a modified version of the QuEChERS (for Quick, Easy, Cheap, Effective, Rugged and Safe) protocol [24]. In brief, vegetative samples (1–5 g pollen/synandria, 5 g female flower base, 15 g whole chopped plant) were combined with water to a final volume of 15 mL. To this sample was added 100 ng of isotopically labeled (d-4) imidacloprid (Cambridge Isotope Laboratories) as an internal standard. The samples were combined with 15 mL of acetonitrile, 6 g magnesium sulfate and 1.5 g sodium acetate. After shaking and centrifuging, 10 mL of the supernatant was combined with 1.5 g magnesium sulfate, 0.5 g PSA, 0.5 g C-18 silica and 2 mL toluene. The samples were shaken and centrifuged and 6 mL of the supernatant was concentrated to 1 mL for instrumental analysis.

#### Analysis

Extracts were analyzed with liquid chromatography/mass spectrometry/mass spectrometry (LC/MS/MS). In 2009, the LC system was an Agilent 1100 LC; 6 µL of the extract was injected onto a Zorbax SB-C18, 2.1×150 mm, 5 micron column. The column is gradient eluted at 0.25 mL per minute from 12.5% methanol in water to 100% methanol. Both solvents have 0.1% formic acid added. In 2010 the LC system was an Agilent 1200 Rapid Resolution system with a Zorbax SB-C18 Rapid Resolution HT 2.1×50 mm, 1.8 micron column using a 3 ul injection with the gradient going from 5% methanol in water to 100% methanol at 0.45 mL/min. In both years, the LC was coupled to a Thermo-LTQ, a linear ion trap mass spectrometer. The system is operated in the positive ion electrospray mode, with a unique scan function for each compound allowing for MS/MS monitoring. Metabolites of imidacloprid (5-hydroxy imidacloprid; imidacloprid urea) and thiamethoxam (clothianadin) were also monitored. The specific parent and product ions monitored for each compound are listed in Table S2 of the supplementary material. Using these extraction and analysis conditions in spiked control samples the compounds averaged 95±18% recovery with detection limits ranging from 0.5 to 2 PPB depending on matrix and the amount of sample available.

### Statistical Analysis

Effects of application method were analyzed for each year and for each of the pesticides and metabolites using an analysis of variance, including blocks in the model [25]. Results for nectar were not analyzed statistically because samples were pooled over blocks in order to have enough material for chemical analysis.

**Table 2:** Neonicotinoid insecticide residues observed in 2010 in various tissues of summer squash after application either to the seed hole just before planting (Soil) or to the transplanted plant through drip irrigation (Drip).

	Imidacloprid (ppb ± SD)		Thiamethoxam (ppb ± SD)	
Tissue	Soil	Drip	Soil	Drip
Female Flower Bases	28±10	15±2	26±12	13±3
Synandria	9±1	11±3	29±22	14±6

## Results

Both imidacloprid and thiamethoxam were detected in all parts of the squash. Data for whole plants and flower parts for each year are presented in Tables 1 and 2, and the data for nectar and pollen are summarized over both years in Table 3. As expected, higher concentrations were observed in the whole plants than in the flower parts, pollen or nectar. Two metabolites of imidacloprid (5-OH imidacloprid and imidacloprid urea) and one metabolite of thiamethoxam (clothianidin) were also detected in whole plant samples. In 2009, when the two application methods were compared in the cultivar “Multipik,” the concentrations of imidacloprid and the two metabolites and the concentration of thiamethoxam and the metabolite clothianidin were significantly higher in whole plant tissue in the drip irrigation treatment than in the soil treatment (df for all tests  = 1,2; imidacloprid: F = 58.386, p = 0.017; 5-OH imidacloprid: F = 27.106, p = 0.035; imidacloprid urea: F = 30.439, p = 0.031; thiamethoxam: F = 79.6, p = 0.012; clothianidin: F = 23.253, p = 0.040). Also in 2009, the concentration of imidacloprid was significantly higher in the synandria (df = 1,2; F = 411.857; p = 0.002) and thiamethoxam was significantly higher in the base of female flowers (df = 1,2; F = 26.518, p = 0.036) in the drip than in the soil treatment. No other comparisons in 2009 between application methods were significantly different.

In 2010, the whole plant tissue was not monitored as the focus was movement of the pesticides into flower parts and then into pollen and nectar. The data for the 2010 yellow summer squash cultivar “Sunray” are presented in Table 2. There were no significant differences between the application methods during this year (for imidacloprid in female flower parts: df = 1,2, F = 4.646, p = 0.164; synandria: df = 1,2; F = 1.240, p = 0.381; pollen, df = 1,3, F = 82.561, p = 0.116; for thiamethoxam in female flower parts: df = 1,2; F = 5.128, p = 0.152; synandria: df = 1,2, F = 2.469, p = 0.257; pollen, df = 1,3, F = 0.586, p = 0.500). Although the data did not rise to the level of significance, the trend in 2010 was for the residues to be higher in the soil treatment than in the drip irrigation treatment for imidacloprid in the female flower bases and thiamethoxam in both the synandria and female flower bases – this trend was the reverse of the 2009 data.

Table 3 presents a summary of the concentrations found in nectar and pollen across years, treatments, and varieties, including the winter squash variety “Bush Delicata.” There were no significant differences in pesticide concentration in pollen with treatment in either year. All samples from treated plants across all three cultivars sampled over the two year period had concentrations of imidacloprid and thiamethoxam in both nectar and pollen greater than 4 ppb. Residues ranged between 5 and 35 ppb for pollen in 12 samples for each insecticide and 5 and 20 ppb for nectar in 6 samples for each insecticide. Averaging across the varieties and years gives overall mean pesticide concentrations in these matrices after insecticide use at labeled rates. In pollen, 14±8 ppb of imidacloprid and 12±9 ppb of thiamethoxam were detected, while in nectar 10±3 ppb of imidacloprid and 11±6 ppb of thiamethoxam were detected.

**Table 3:** Summary of neonicotinoid measurements in pollen and nectar of squash, combining all treatments, years, and varieties.

	Imidacloprid (ppb)		Thiamethoxam (ppb)	
	Pollen	Nectar	Pollen	Nectar
Mean concentration (± SD)	14±8	10±3	12±9	11±6
Number of samples	12	6	12	6
Minimum concentration	6	5	5	5
Maximum concentration	28	14	35	20

## Discussion

In assessing the potential hazard of neonicotinoid insecticides to pollinators, two kinds of data are required: 1) levels of exposure and 2) effects of exposure at those levels on the biology of the pollinators. Past risk assessments have based their assumptions about levels of exposure on concentrations of neonicotinoids found in nectar and pollen of crops treated as seeds because those were the only data available at the time. Rortais et al. [26] and Halm et al. [27] used 3.4 ppb of imidacloprid for pollen and 1.9 ppb for nectar as maximum levels, and Cresswell [28] considered 0.7–10 ppb to be the field-realistic range of concentration of imidacloprid in nectar. Cresswell [28] noted that “more studies of the amounts of neonicotinoids in nectar and pollen are needed to establish the field-realistic range because the available data is meager.”

Our results partially confirm those of Dively and Kamel [17] in expanding the range of concentration of neonicotinoids found in nectar and pollen in the field. Of the treatments Dively and Kamel used, their “transplant-drip” treatment is the most similar to our treatments, and had similar levels of concentration of neonicotinoids in nectar. Our levels of concentration of neonicotinoids in pollen were similar to the levels they found in 2010, although the levels they found in 2009 were 6–7X higher than ours. They also had higher levels of metabolites in both nectar and pollen than we found (Data not shown here). They also were able to test a wider range of metabolites.

The differences in concentrations between application methods we observed in both male and female flower parts in 2009 were not repeated in 2010, perhaps due to differences in weather or crop varieties. Dively and Kamel [17] also had no consistent significant differences when comparing application in transplant water, through drip irrigation, and by foliar spray, although they had significantly lower concentrations in nectar and pollen when imidacloprid was applied in a drench to bedding plants before transplant and when thiamethoxam was applied as a seed treatment.

One reason for the higher concentration of neonicotinoids in nectar and pollen with soil or drip application compared to crops treated as seeds may be because the labeled rates of neonicotinoid applied per unit area are higher for the application methods we used. The highest rate we found for a seed treatment with imidacloprid, for corn in Northern Europe - 95 g AI/ha, [29], was one-third the lowest labeled rate for soil application of imidacloprid on squash, 281 g AI/ha [30] and 27% of the lowest rate of imidacloprid used in this experiment (358 g AI/ha). The seed treatment tested by Dively and Kamel [17], not yet available to us in Connecticut, uses thiamethoxam at 0.75 mg AI per seed. At recommended seeding rates for pumpkin, that would be 13 g AI/ha or 9% of the rate used here.

What would be the effects of the concentrations measured here on the exposure of honey bees and other bees? The concentrations of imidacloprid and thiamethoxam found in nectar are particularly important because honey bees consume far more sugar (as nectar or processed into honey) than pollen over their lifespan. Each worker bee during the summer, going through all the stages of development and a succession of house bee and foraging tasks, consumes 736–1575 mg. of sugar, while each worker bee surviving over the winter consumes an additional 792 mg of sugar maintaining the temperature of the hive [26]. The estimated pollen consumed per bee (stored as bee bread, and processed by nurse bees into glandular secretions for feeding to bee larvae) is only 70.4 mg [26]. Since *C. pepo* nectar is 28–42% sugars by weight [19], each worker bee would consume a minimum equivalent of 1750 mg of nectar over a summer lifespan. The extent to which imidacloprid and thiamethoxam are broken down when pollen and nectar are processed and stored as bee bread and honey is unknown.

A number of studies have been conducted on the sublethal effects of imidacloprid on honey bees. Cresswell [28] did a meta-analysis of 13 studies feeding imidacloprid to honey bee colonies in sugar water (50% sucrose) and modeled the reduction in honey bee colony performance that would be predicted at sublethal doses that have been found in field studies, including the range of doses found here. In addition, recent studies have found interactions of sublethal concentrations of neonicotinoids with honey bee immune systems and with the pathogen *Nosema ceranae* causing increased mortality of honey bees at concentrations of 0.7 and 7 ppb in sugar water [31] or 5 ppb in pollen [32].

There is much less information available on sublethal effects of pesticides on other species of bees. Whitehorn et al. [33] found that *Bombus terrestris* (a European species of bumble bee) had an 85% reduction in queen production over the season when fed imidacloprid at concentrations of 0.7 ppb in sugar water and 6 ppb in pollen for two weeks before being placed in the field.

Both honey bees and bumble bees are generalist feeders on a very wide range of other pollen and nectar sources in addition to *Cucurbita*, so their actual feeding exposure to neonicotinoids would depend on the range of alternative food sources available in addition to treated crop plants. However, squash bees are specialists on *Cucurbita*, feeding their larvae exclusively on *Cucurbita* pollen [22], and also build their nests in soil, often directly beneath squash and pumpkin vines [21], so they could have much more exposure to the soil-applied insecticides used on these crops.

There is much research still to be done on modes of exposure of bees to pesticides [14], [34], and effects of pesticides on bees [16]. Very little research has been done on fruit and vegetable crops like squash, which are frequently treated with insecticides, and which are entirely dependent on pollination by bees in order to set fruit and produce a yield.

## Supporting Information

Table S1 Chronology of planting, treatments and sampling.
(DOCX)

Table S2 MS/MS transitions monitor.
(DOCX)
