# Supplementary material for: Movement of Soil-Applied Imidacloprid and Thiamethoxam into Nectar and Pollen of Squash (Cucurbita pepo)
Source: PLoS One. 2012 Jun 27;7(6):e39114. doi: 10.1371/journal.pone.0039114 (PMC3384620; doi:10.1371/journal.pone.0039114)
Supplement: Table S1 — Chronology of planting, treatments and sampling. (DOCX) [file pone.0039114.s001.docx]

Table S1. Chronology of planting, treatments and sampling

| Action | Date in 2009 | Date in 2010 |
| --- | --- | --- |
| Application of insecticides by spraying soil in seed holes (treatments 2 & 3) | 4 June | 14 June |
| Planting seeds in the field (treatments 1, 2 & 3) | 5 June | 15 June |
| Starting seeds in greenhouse (treatments 4 & 5) | 1 June | 16 June |
| Transplanting (treatments 4 & 5) | 17 June | 28 June |
| Application of insecticides through drip irrigation to transplants (treatments 4 & 5) | 22 June | 1 July |
| Collection of female flower bases | 13 July | 16 July, 26 July |
| Collection of male synandria | 14 July | 20 July – 3 Aug. |
| Collection of whole plant samples | 15-16 July | Not done |
| Collection of male flowers for pollen | 22 July, 29 July | 29 July – 11 Aug. |
| Collection of nectar from female flowers | 29 July – 5 Aug. | 21 July – 6 Aug. |
