# Supplementary material for: Movement of Soil-Applied Imidacloprid and Thiamethoxam into Nectar and Pollen of Squash (Cucurbita pepo)
Source: PLoS One. 2012 Jun 27;7(6):e39114. doi: 10.1371/journal.pone.0039114 (PMC3384620; doi:10.1371/journal.pone.0039114)
Supplement: Table S2 — MS/MS transitions monitor. (DOCX) [file pone.0039114.s002.docx]

Table S2. MS/MS transitions monitored

| Compound | Parent Ion m/z | Product Ion m/z |
| --- | --- | --- |
| D4 - Imidacloprid | 260 | 179, 213, 214, 216 |
| Imidacloprid | 256 | 175, 209, 210, 212 |
| Hydroxy-imidacloprid | 272 | 190, 228 |
| Imidaclopriod-Urea | 212 | 128 |
| Thiamethoxam | 292 | 211, 246 |
| Clothianadin | 250 | 132, 168, 169 |
